# Exposure of Mice to Thirdhand Smoke Modulates In Vitro and In Vivo Platelet Responses

**DOI:** 10.3390/ijms23105595

**Published:** 2022-05-17

**Authors:** Daniel Villalobos-García, Hamdy E. A. Ali, Ahmed B. Alarabi, Medhat S. El-Halawany, Fatima Z. Alshbool, Fadi T. Khasawneh

**Affiliations:** 1 Department of Pharmaceutical Sciences, Irma Lerma Rangel College of Pharmacy, Texas A&M University, Kingsville, TX 78363, USA; danielvg@tamu.edu (D.V.-G.); haali@tamu.edu (H.E.A.A.); m.elhalawany@tamu.edu (M.S.E.-H.); 2 Department of Pharmacy Practice, Irma Lerma Rangel College of Pharmacy, Texas A&M University, Kingsville, TX 78363, USA; alarabi@tamu.edu (A.B.A.); falshbool@tamu.edu (F.Z.A.)

**Keywords:** thirdhand smoke, platelet, thrombosis, hemostasis, tobacco

## Abstract

Smoking is a risk factor for a variety of deleterious conditions, such as cancer, respiratory disease and cardiovascular disease. Thrombosis is an important and common aspect of several cardiovascular disease states, whose risk is known to be increased by both first- and secondhand smoke. More recently, the residual cigarette smoke that persists after someone has smoked (referred to as thirdhand smoke or THS) has been gaining more attention, since it has been shown that it also negatively affects health. Indeed, we have previously shown that 6-month exposure to THS increases the risk of thrombogenesis. However, neither the time-dependence of THS-induced thrombus formation, nor its sex dependence have been investigated. Thus, in the present study, we investigated these issues in the context of a shorter exposure to THS, specifically 3 months, in male and female mice. We show that the platelets from 3-month THS-exposed mice exhibited enhanced activation by agonists. Moreover, we also show that mice of both sexes exposed to THS have decreased tail bleeding as well as decreased thrombus occlusion time. In terms of the role of sex, intersex disparities in thrombus development and hemostasis as well as in platelet aggregation were, interestingly, observed. Together, our findings show that exposing mice to THS for 3 months is sufficient to predispose them to thrombosis; which seems to be driven, at least in part, by an increased activity in platelets, and that it does not manifest equally in both sexes.

## 1. Introduction

Tobacco smoking causes a host of conditions, including cancer, pulmonary disease, arthritis and osteoporosis [1]. Moreover, smoking is the leading cause of cardiovascular disease (CVD), causing up to approximately 1/3 of associated deaths [2], with CVD being the leading cause of death worldwide. In light of this, the mechanisms underlying smoking-mediated increases in the risk of CVD have been under investigation. For example, smoking has been shown to increase the risk of atherosclerosis, causing the vascular lumen to be narrower and more prone to ulceration [2]. Additionally, it has been found that tobacco products also modulate platelet function [2,3,4] which—combined with the development of atherosclerosis—can drastically increase the risk of acute thrombosis [2,5]. Thus, research has shown that exposure to tobacco smoke induces the priming of platelets, leading to a more pronounced response after stimulation [6,7,8,9]. Subsequent studies showed that this effect is not limited to just tobacco products, but also to waterpipes [10], electronic cigarettes [11,12] and even nicotine itself [6,10], suggesting that the alkaloid is at least in part directly responsible for this effect.

Decades of research on the health effects of tobacco smoking have firmly established that the myriad of deleterious effects it causes are not just associated with firsthand smoke (FHS), but also with secondhand smoke (SHS) [13]. To this end, the health effects that tobacco smoke exert on nonsmokers have recently gained renewed interest with the discovery of thirdhand smoke (THS), which is a term coined to refer to the particles of tobacco smoke that are left on indoor surfaces, such as clothes, toys, furniture and carpets [14]. Hence, THS is composed of a myriad of molecules/toxicants—such as nicotine, cotinine and myosmine—that not only can affect the air quality, but also accumulate overtime, generating (more) toxic byproducts by chemical reaction with oxygen and other elements [14]. In fact, these particles could become more harmful as they “age” [14]. Moreover, exposure to THS can be through multiple routes, including inhalation, skin contact or ingestion [14]. This is of great importance as it puts vulnerable populations such as infants/children at a greater risk of becoming exposed unintentionally [14].

To this end, THS exposure has been linked to many harmful effects, including DNA damage, steatosis and hyperglycemia, making THS exposure a potential health risk/concern [14]. Strikingly, we have previously shown that chronic/long term THS exposure (6 months) increases the risk of thrombosis [15,16], as is the case with FHS and SHS [2]. However, it is important to examine the time course of these effects and determine whether THS effects would manifest under shorter exposure. Additionally, whether sex has an effect on this phenotype is yet to be investigated. In this manuscript, we show that shorter exposure, i.e., 3 months, to THS not only enhanced platelet function but also accelerated hemostasis and thrombosis. Furthermore, while this increased platelet reactivity was visible in both males and females, intersex differences in thrombus formation, hemostasis and platelet aggregation were noticeable when males’ and females’ data were compared. Taken together, our findings show that THS-mediated negative health effects, at least in the context of occlusive disorders, do not always necessitate long-term exposures to be observed, and they are no less relevant than those from FHS and SHS, with some of these effects being sex-dependent.

## 2. Results

### 2.1. Platelets from THS-Exposed Mice Exhibit Increased Aggregation and Dense Granule Secretion

**Figure 1:**
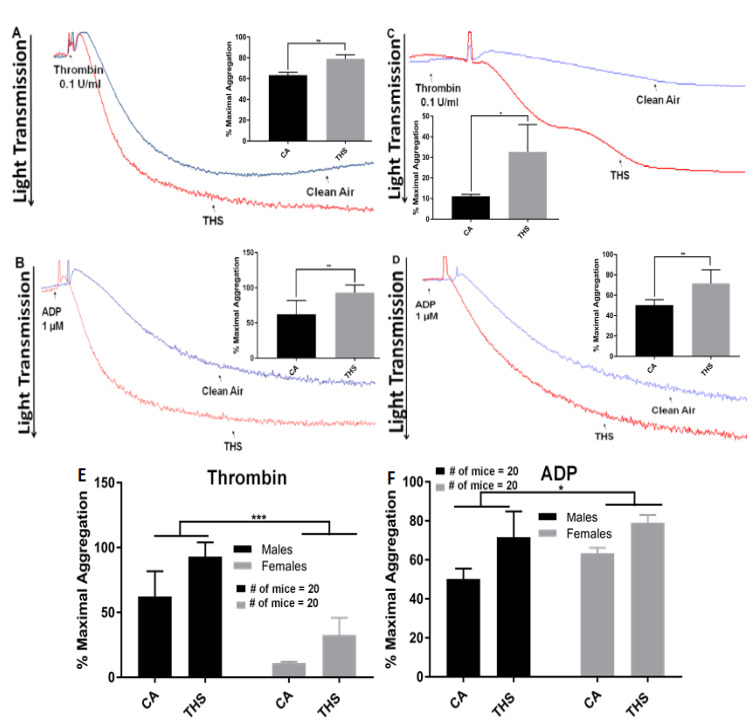
THS-exposed platelets show increased aggregation. THS-exposed and CA-exposed platelets from male (**A**,**B**) and female (**C**,**D**) mice were stimulated with either (**A**,**C**) thrombin (0.1 U/mL) or (**B**,**D**) ADP (1 μM). Intersex differences for thrombin are shown in (**E**) whereas those for ADP are shown in (**F**). Each experiment was repeated four times with blood pooled from at least five THS-exposed and CA-exposed mice (* *p* < 0.05; ** *p* < 0.01; *** *p* < 0.001).

**Figure 2:**
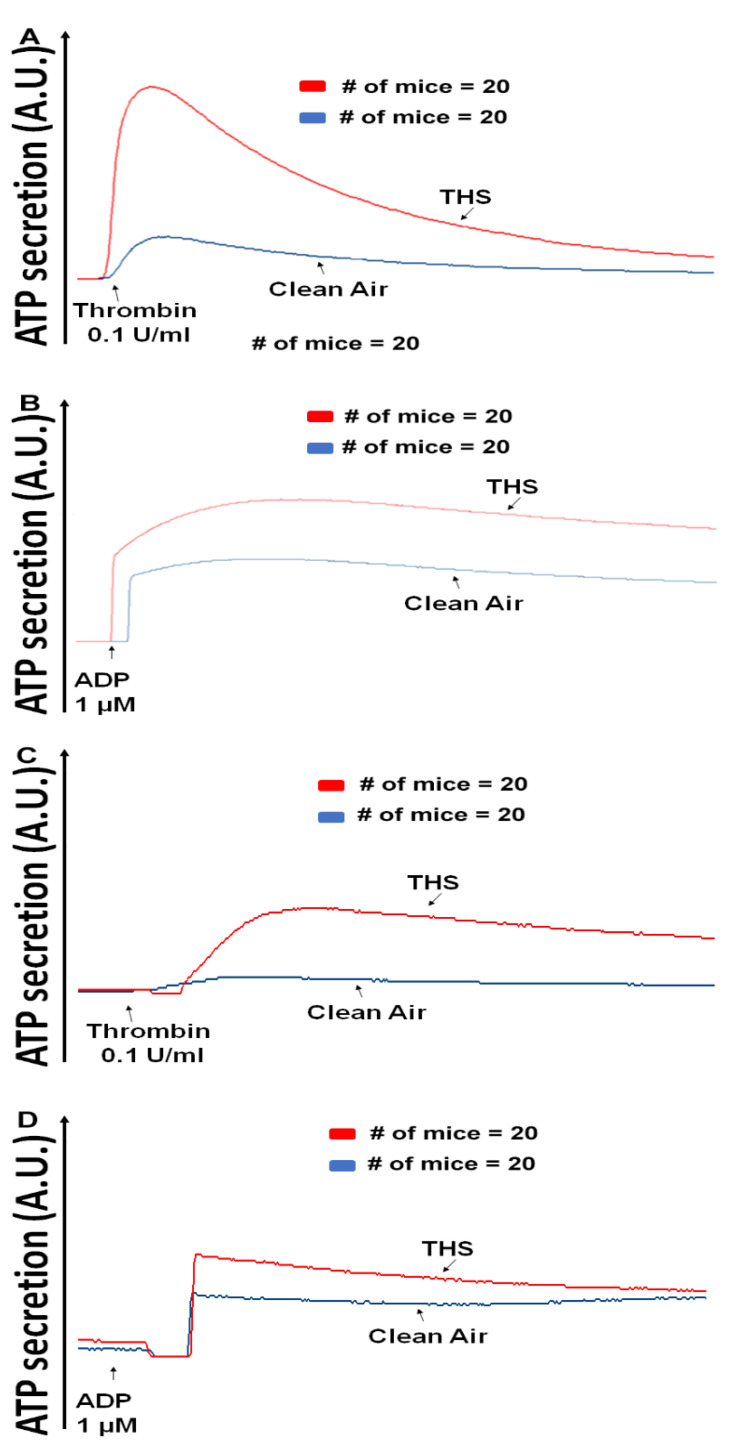
THS-exposed platelets show increased dense granule secretion. THS-exposed and CA-exposed platelets from male (**A**,**B**) and female (**C**,**D**) mice were stimulated with either (**A**,**C**) thrombin (0.1 U/mL) or (**B**,**D**) ADP (1 μM). Each experiment was repeated four times with blood pooled from at least five THS-exposed and CA-exposed mice.

To assess whether a shorter exposure to THS, specifically 3 months would produce effects on platelets, we first studied their aggregation response, since this assay is the gold standard for platelet function evaluation [17]. When using the agonists thrombin (Figure 1A and Figure 1C; male and female, respectively) and ADP (Figure 1B and Figure 1D; male and female, respectively), we observed that platelets from THS-exposed mice did indeed exhibit a significant increase in aggregation compared to the controls. Indeed, this enhanced aggregation of platelets from THS-exposed mice was pronounced when either males or females were compared with their CA-exposed counterparts (Figure 1A–D). Comparing platelet aggregation between both sexes revealed a significant difference (*p* = 0.037) between males and females, with male platelets being slightly more sensitive to THS exposure than female platelets (Figure 1E,F). Mirroring the aggregation results, dense granule release was also elevated in platelets from THS-exposed male and female mice regardless of the agonist used (Figure 2A–D).

### 2.2. Platelets from THS-Exposed Mice Exhibit Increased Integrin αIIbβ3 Activation, α-Granule Secretion and Phosphatidylserine Expression

**Figure 3:**
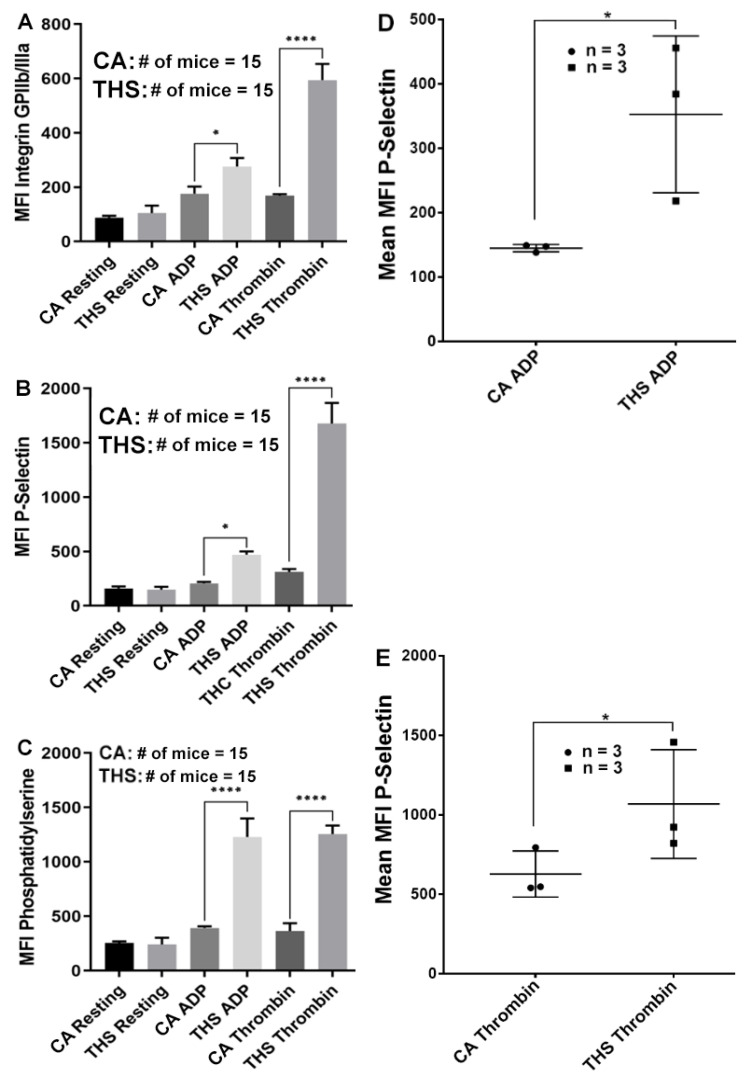
THS-exposed platelets show increased integrin αIIbβ3, P-Selectin and phosphatidylserine expression. THS-exposed and CA-exposed platelets were stimulated with either thrombin (0.1 U/mL) or ADP (1 μM) for 5 min, and processed for (**A**) integrin αIIbβ3, (**B**,**D**,**E**) P-Selectin, (**C**) phosphatidylserine expression as described in the Methods section. Results are expressed as the mean fluorescence intensity (MFI) or mean of the MFI (**D**,**E**) by flow cytometry. Each experiment was repeated three times with blood pooled from at least five THS-exposed and CA-exposed mice ((**A**–**C**); * *p* < 0.05; **** *p* < 0.0001) or with blood from a single mouse for a total of three mice ((**D**,**E**); * *p* < 0.05).

In the following series of experiments, and after documenting an increase in aggregation and dense granule secretion responses, we tested if the 3-month exposure to THS would also result in an increase in other platelet activation markers. By employing flow cytometry analysis, we observed that α_IIb_β_3_ activation, α-granule secretion and phosphatidylserine expression are all potentiated in response to ADP and thrombin in the THS platelets (Figure 3A, Figure 3B and Figure 3C, respectively) compared to the controls. These results are consistent with the increased aggregation and secretion response previously shown. Together, these findings support the notion that platelets have become hyperactive after exposure to THS for three months. Of note, when agonist-induced α-granule secretion was measured using platelets from a single mouse (females were tested herein), secretion was again found to be enhanced (Figure 3D,E), with no apparent differences observed in their resting levels.

### 2.3. THS-Exposed Mice Have a Shortened Tail Bleeding Time

**Figure 4:**
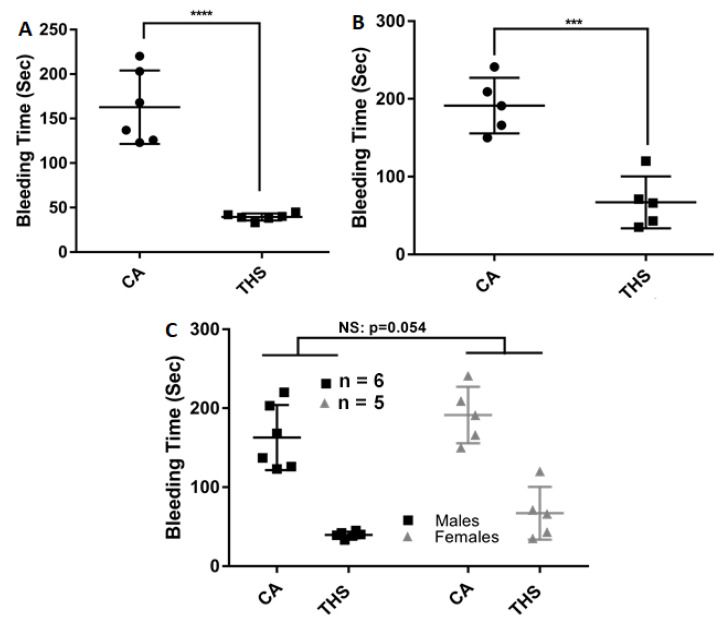
THS-exposed mice showed decreased tail bleeding times. THS-exposed and CA-exposed (**A**) male and (**B**) female mice were anesthetized with isoflurane and a 5 mm segment from their tails was transected and the tail bleeding assay was performed as described in the methods section. Intersex differences are shown in panel (**C**). Each point represents the bleeding time of a single animal (*** *p* < 0.001; **** *p* < 0.0001; NS: none significant).

As the effects of THS on platelet activity became clear under in vitro conditions, we next sought to determine whether these effects would also manifested in vivo. To address this issue, we first conducted the tail bleeding assay and determined the time it takes for the bleeding to stop. As can be seen in Figure 4A (males) and Figure 4B (females), exposure to THS for 3 months drastically reduced the bleeding time compared to the control, suggesting that the hyperactivity of platelets has an impact in vivo. Males in the clean air group bled for an average of [162.8 ± 16.85] seconds compared to [39.5 ± 1.648] seconds in the THS group (Figure 4A). As for the average bleeding time in females, it was [191.4 ± 16.01] seconds in the clean air group and [67 ± 14.88] seconds in the THS group (Figure 4B). Additionally, we compared bleeding in males and females to assess if there are any sex-related differences. Although females’ average bleeding duration was longer than males’ both in CA- and THS-exposed, the difference was only marginally significant, with a *p* value of 0.054 (Figure 4C).

### 2.4. THS-Exposed Mice Have a Shortened Thrombosis Occlusion Time

**Figure 5:**
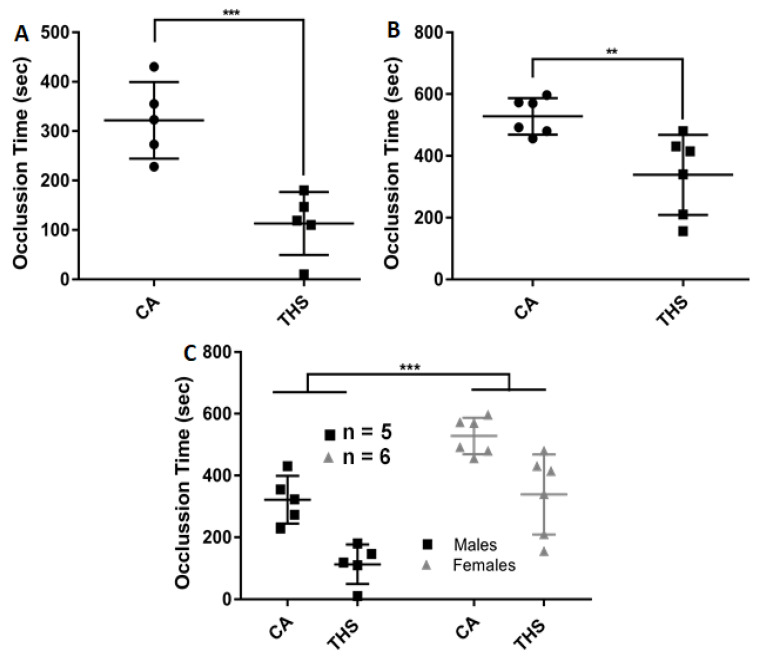
THS-exposed mice showed decreased occlusion times. THS-exposed and CA-exposed (**A**) male and (**B**) female mice were anesthetized with avertin and carotid injury-induced thrombosis was induced with a FeCl_3_ solution as described in the methods section. Intersex differences are shown in panel (**C**). Each point represents the occlusion time of a single animal (** *p* < 0.01; *** *p* < 0.001).

Since chronic cigarette smoking is a well-known risk factor in the development of thrombotic events [5], and exposure to THS for 6 months increased the risk of thrombogenesis [15], we determined if a shorter THS exposure, namely 3 months, has any effects in this regard. We employed the FeCl_3_ induced thrombosis model to measure the time required for an arterial occlusion to occur in both male and female mice exposed to THS, relative to their control counterparts. As shown in Figure 5A, THS-exposed male mice were more prone to occlusion than the clean air-exposed controls, shortening the time from [321.8 ± 34.63] seconds to only [113.2 ± 28.55] seconds. Females exposed to THS similarly displayed a reduction in occlusion, with [528 ± 24] seconds in CA-exposed females compared to [338.7 ± 52.99] seconds in THS-exposed females (Figure 5B). Interestingly, when male and female responses to THS exposure were compared, a substantial sex-based variation in occlusion time was observed (Figure 5C). These findings corroborate the shorter bleeding time observed in these mice, indicating that THS exposure has a pro-thrombotic effect in vivo.

## 3. Discussion

While the inhalation of first- and second-hand smoke (FHS and SHS) is a short-lived and “direct” way of exposure, the exposure to THS can go on for months after the smoking ceases. For example, long-term smoking in a casino was found to create deep THS reservoirs that persisted for months after a smoking ban [18]. Moreover, in terms of THS exposure route, it was found to also include dermal absorption by touching contaminated surfaces and even ingestion [14]. Even though there is a decrease in smoking, (and smoking is not allowed in public places), 88 million nonsmokers 3 years of age or older living in the homes of smokers are exposed to THS [19,20] in the US. Therefore, it is important to investigate how “quickly” the exposure to THS can lead to significant health effects. To this end, previous studies have shown that some of the in vivo effects of THS in mice—such as delays in wound healing, hypertriglyceridemia and hyperglycemia—arise within 6 months after the exposure begins [14,21]. Moreover, we have also previously showed that exposing mice to THS for 6 months predisposes them to thrombosis, with the mice exhibiting shorter bleeding and occlusion times as well as an increased platelet response to agonists compared to the controls [15]. However, many of the health effects caused by tobacco-related products and e-cigarettes can become apparent very shortly after exposure begins [7,11,22], a notion that warrants investigation in the context of THS. It is important to note that it is thought that THS could potentially be more toxic than fresh SHS [14,19,23,24], and this notion is consistent with internal research findings by Philip Morris [25], which were made public due to settlements of litigation [23]. It is also to be noted that tobacco toxicants/metabolites persist in humans and animals for months or even years [26,27,28,29,30,31,32,33,34,35,36,37].

Herein, we show that the effects that THS exerts on platelets and hemostasis in mice can manifest in as short a timeframe as 3 months. Indeed, we found that the shortened tail bleeding and occlusion times are comparable to those reported in previous studies regarding tobacco products [10,11], suggesting that these short-term hematological effects that THS has in vivo may be as significant as those caused by FHS and SHS. Likewise, our results confirm that THS exposure primes platelets, showing a more robust activation when they are stimulated with agonists. Thus, THS was found to enhance a host of platelet functional responses, such as aggregation, secretion, integrin activation and phosphatidylserine exposure. The latter findings are consistent with the phenotype observed in vivo. These in vivo and in vitro responses were clearly evident in both sexes. Interestingly, intersex comparisons revealed significant differences in male and female responses to THS exposure, which manifested both in vitro and in vivo, and was also documented in our previously reported in utero THS studies [16]. To this end, our data suggests that male mice are more sensitive to the effects of THS exposure than females. Indeed, there are important sex disparities in the pathological effects of smoke on males and females. For instance, and consistent with our observation, it was found that SHS exposure in rats, during the neonatal to adolescent period, increased infarct size in males more than in females [38]. Furthermore, thrombin-mediated platelet activation is enhanced in males and diminished in females during myocardial infarction in both mice and humans [39]. Contrary to this notion, it was previously shown that the risk of myocardial infarction associated with cigarette smoking is higher in women than in men [40].

Collectively, our data show, for the first time, that 3 months of THS exposure are sufficient to induce platelets to become hyperactive, which in turn increases the risk of developing thrombosis. These data support the notion that a relatively shorter duration of THS exposure, 3 months relative to the 6 months we previously showed [15], has the capacity to increase the risk of thrombogenesis; underscoring the potential dangers of exposure to THS. These findings should be reconciled with the fact that a large portion of our population is not aware of THS existence [41,42,43]. These results should also inform policy and smoking cessation efforts to limit exposure to tobacco smoke.

## 4. Materials and Methods

### 4.1. Reagents and Materials

Thrombin, ADP and luciferase were bought from Chrono-Log Corporation (Havertown, PA, USA). FeCl_3_ was obtained from Sigma-Aldrich (St. Louis, MO, USA). FITC-conjugated anti-P-selectin and FITC-conjugated annexin V were purchased from BD Biosciences (Franklin Lakes, NJ, USA), while PE-conjugated Jon/A was purchased from Emfret Analytics (Würzburg, Germany). Research cigarettes (3R4F) were purchased from the University of Kentucky.

### 4.2. Animals

C57BL/6 mice were obtained from Jackson Laboratory (Bar Harbor, ME, USA). Mice were housed in groups of 1–4 at 24 °C, under 12/12 light/dark cycles, with access to water and food ad libitum. Mice were divided into clean air (control) and THS exposed groups. The THS group was exposed to THS for 3 months, whereas the control group was exposed to clean air for the same period. All experiments involving animals were performed in compliance with the institutional guidelines, and were approved by the Institutional Animal Care and Use Committee.

### 4.3. THS Exposure

Mice were exposed to THS as previously described [15]. Briefly, household fabrics (carpet, curtain material and upholstery) were exposed to cigarette smoke by placing them inside a smoking exposure apparatus (Teague Enterprises, Woodland, CA, USA). The exposure lasted for 3 months and consisted of 40 cigarettes/day per for 1 week, with the smoke being directed into two exposure chambers where the materials were placed. At the end of each week, the material is placed in the cages was replaced with freshly exposed material, and this cycle repeated for 4 to 5 weeks before the material is discarded. We employed two sets of material that were exposed on an alternating-week basis. This approach ensures that mice are constantly exposed to THS and that they are subjected to material that has been exposed not only to fresh but also aged THS, thereby mimicking real-life exposure conditions.

### 4.4. Platelet Preparation

Platelets were prepared as previously described [15,16]. Mice were anesthetized with isoflurane and blood was collected from the heart with a syringe containing a sodium citrate solution (3.8%). Platelet-rich plasma (PRP) was obtained by centrifuging the blood at 180× *g* for 10 min at room temperature. Platelets were then counted with an automated hematology analyzer (Drew Scientific Hemavet). Washed platelets were prepared by centrifuging PRP at 483× *g* for 10 min. The pellet was resuspended with HEPES-buffered saline (pH 6.5) containing 1 mM EGTA, 0.37 U/mL of apyrase and 10 ng/mL of PGI_2_. The platelets were finally washed and resuspended with HEPES-buffered saline (pH 7.4).

### 4.5. Platelet Aggregation

PRP was adjusted with HEPES-buffered saline to 70,000 platelets/μL in a final volume of 250 μL for each reading. Platelets were activated with either ADP (1 μM) or thrombin (0.1 U/mL) and aggregation and dense granule secretion (ATP release) were measured by light transmission aggregometry and the luminescence method, respectively, using a model 700 aggregometer (Chrono-Log Corporation; Havertown, PA, USA).

### 4.6. Flow Cytometry

Flow cytometric analysis was performed with washed platelets as previously described [15,16,44] or using washed platelets isolated from a single mouse. Briefly, washed platelets (2 × 10^8^) were stimulated with either ADP (1 μM) or thrombin (0.1 U/mL) and incubated for 5 min. The reaction was then stopped with 0.5% formaldehyde for 30 min at room temperature. Next, the fixed platelets were incubated with either PE-conjugated Jon/A antibody, FITC-conjugated anti-P-selectin antibody, or FITC-conjugated annexin V for 30 min at room temperature. Finally, the platelets were diluted 2-fold with PBS (pH 7.4). Flow cytometry and data analysis were carried out using BD Accuri C6 plus flow cytometer system (BD Biosciences; Franklin Lakes, NJ, USA). Data obtained from single mice is presented as mean of the MFI of at least replicates obtained from a total of three female mice.

### 4.7. Tail Bleeding Time Assay

The tail bleeding assay was performed as previously described [11,15,16]. Briefly, mice were anesthetized with isoflurane and placed on an isothermic blanket (37 °C). Then the tail was transected 5mm from the tip with a scalpel and immediately submerged into a warm saline solution, that is maintained at a constant 37 °C temperature). The bleeding time was measured until it completely stopped. However, to avoid excessive bleeding, the experiment was stopped after 10 min, even if the bleeding did not stop, with “10 min” considered the cutoff for statistical analysis. Of note, bleeding stoppage was not considered unless it lasted for at least 1 min.

### 4.8. Carotid Artery Injury–Induced Thrombosis Model

The thrombosis model was performed as previously described [11,15,16]. Briefly, mice were anesthetized with avertin (2.5%) at a dose of 250 mg/kg and the carotid artery was exposed, cleaned with saline solution (37 °C) and isolated. The baseline blood flow was then measured with a Transonic Micro-Flowprobe (Transonic Systems Inc, Ithaca, NY, USA). Once the blood flow is stabilized, a filter disc paper (1 mm wide) soaked in 7.5% FeCl_3_ was placed on top of the artery for 3 min. The blood flow was then continuously monitored until a stable occlusion formed (no blood flow for 2 min). The time of occlusion formation was calculated as the difference in time between stable occlusion and removal of filter paper, with 20 min considered the cutoff for statistical analysis.

### 4.9. Platelet Count

Platelet count was determined using a automated hematology analyzer (Drew Scientific, Dallas, TX, USA).

### 4.10. Statistical analysis

As applicable, experiments were performed at least three times with pooled blood from at least five mice each time or from blood obtained from a single mouse. Data analysis was achieved using the statistical software GraphPad PRISM (San Diego, CA, USA) and presented as mean ± SEM. One-way ANOVA was used for the analysis of flow cytometry and aggregation data, while the Mann–Whitney test was used for the occlusion and bleeding data, as well as single mouse flow cytometry data. Statistical significance was accepted at *p* < 0.05.

## 5. Conclusions

In conclusion, our study provides evidence that exposure to THS for 3 months modulates platelet function in vitro and in vivo in a sex-specific fashion, and support the notion that it is detrimental to health. It also underscores the importance of not underestimating its danger.

## Data Availability

Data will be made available by the corresponding authors upon request.
